# Tris(1-naphth­yl)arsine chloro­form solvate

**DOI:** 10.1107/S1600536809041646

**Published:** 2009-10-17

**Authors:** Omar bin Shawkataly, Imthyaz Ahmed Khan, Chin Sing Yeap, Hoong-Kun Fun

**Affiliations:** a Chemical Sciences Programme, School of Distance Education, Universiti Sains Malaysia, 11800 USM, Penang, Malaysia; b X-ray Crystallography Unit, School of Physics, Universiti Sains Malaysia, 11800 USM, Penang, Malaysia

## Abstract

In the title compound, C_30_H_21_As·CHCl_3_, the dihedral angles between the three naphthalene ring systems [r.m.s. deviations = 0.007, 0.009 and 0.020 Å] are 72.54 (4), 88.05 (4) and 83.36 (4)°. In the crystal, the mol­ecules are stacked down the *a* axis being consolidated by C—H⋯π and π–π inter­actions [centroid to centroid distance = 3.7839 (7) Å].

## Related literature

For general background to tris­(1-naphth­yl)arsine, see: Cullen *et al.* (1995 ▶). For related structures, see: Kamepalli *et al.* (1996 ▶); Shawkataly *et al.* (2009 ▶). For the synthesis, see: Burfield *et al.* (1977 ▶, 1978 ▶); Burfield & Smithers (1978 ▶); Michaelis (1902 ▶). For description of the Cambridge Structural Database, see: Allen (2002 ▶). For the stability of the temperature controller used for the data collection, see: Cosier & Glazer (1986 ▶).
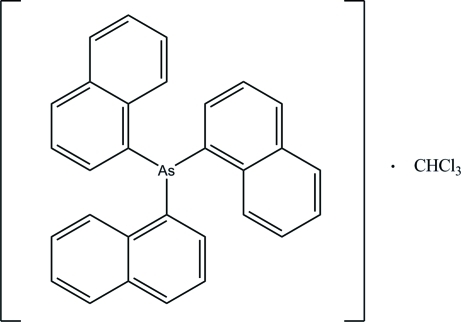


## Experimental

### 

#### Crystal data

C_30_H_21_As·CHCl_3_
*M* *_r_* = 575.76
Triclinic, 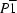

*a* = 9.1326 (2) Å
*b* = 11.9473 (2) Å
*c* = 12.3971 (2) Å
α = 77.432 (1)°
β = 87.455 (1)°
γ = 75.434 (1)°
*V* = 1277.72 (4) Å^3^
*Z* = 2
Mo *K*α radiation
μ = 1.66 mm^−1^
*T* = 100 K
0.62 × 0.23 × 0.10 mm

#### Data collection

Bruker SMART APEXII CCD area-detector diffractometer
Absorption correction: multi-scan (**SADABS**; Bruker, 2005 ▶) *T*_min_ = 0.427, *T*_max_ = 0.849
37994 measured reflections
7382 independent reflections
6791 reflections with *I* > 2σ(*I*)
*R*_int_ = 0.027

#### Refinement

*R*[*F* ^2^ > 2σ(*F* ^2^)] = 0.023
*wR*(*F* ^2^) = 0.061
*S* = 1.04
7382 reflections
316 parameters
H-atom parameters constrained
Δρ_max_ = 0.47 e Å^−3^
Δρ_min_ = −0.29 e Å^−3^

### 

Data collection: *APEX2* (Bruker, 2005 ▶); cell refinement: *SAINT* (Bruker, 2005 ▶); data reduction: *SAINT*; program(s) used to solve structure: *SHELXTL* (Sheldrick, 2008 ▶); program(s) used to refine structure: *SHELXTL*; molecular graphics: *SHELXTL*; software used to prepare material for publication: *SHELXTL* and *PLATON* (Spek, 2009 ▶).

## Supplementary Material

Crystal structure: contains datablocks global, I. DOI: 10.1107/S1600536809041646/tk2554sup1.cif

Structure factors: contains datablocks I. DOI: 10.1107/S1600536809041646/tk2554Isup2.hkl

Additional supplementary materials:  crystallographic information; 3D view; checkCIF report

## Figures and Tables

**Table 1:** Hydrogen-bond geometry (Å, °)

*D*—H⋯*A*	*D*—H	H⋯*A*	*D*⋯*A*	*D*—H⋯*A*
C4—H4*A*⋯*Cg*1^i^	0.93	2.68	3.6013 (14)	169
C14—H14*A*⋯*Cg*2^ii^	0.93	2.86	3.7421 (15)	160

Symmetry codes: (i) 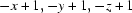; (ii) 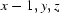. *Cg*1 and *Cg*2 are centroids of the C25–C30 and C5–C10 benzene rings, respectively.

**Fig. 1.**
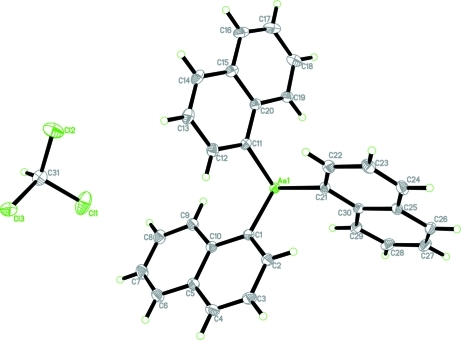
The molecular structure of the title compound with 50% probability ellipsoids for non-H atoms.

**Fig. 2.**
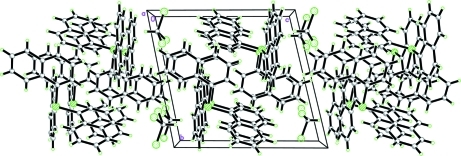
The crystal packing of the title compound, viewed down the a axis, showing the molecules stacked down the a axis.

## supplementary crystallographic information

### Comment

Tris(1-naphthyl)arsine has been used in the synthesis of osmium and ruthenium cluster derivatives (Cullen *et al.*, 1995). A search of the Cambridge Structural Database (Allen, 2002) revealed no structure containing this molecule. Among substituted naphthylarsines, only the structure of tris[8-(dimethylamino)-1-naphthyl]arsine (Kamepalli *et al.*, 1996) is known.

The asymmetric unit of the title compound comprises a molecule of tris(1-naphthyl)arsine and a solvent chloroform molecule (Fig. 1). The As–C bond lengths lie in the range 1.9595 (11) to 1.9635 (12) Å, and the C–As–C angles lie in the range  98.97 (5) to 100.92 (5) °. The values are comparable to those found in related structures (Kamepalli *et al.*, 1996; Shawkataly *et al.*, 2009). The dihedral angles between the three naphthalene ring systems (C1–C10/C11–C20, C1–C10/C21–C30 and C11–C20/C21–C30) are 72.54 (4), 88.05 (4) and 83.36 (4)°, respectively. In the crystal packing (Fig. 2), the molecules are stacked down the *a* axis being consolidated by  C—H···π (Table 1) and π–π interactions [*Cg*1···*Cg*3^iii^ = 3.7839 (7) Å; *Cg*1 and *Cg*3 are centroids of benzene rings C25–C30 and C21–C25/C30, respectively; (iii) -*x*, 2 - *y*, 1 - *z*].

### Experimental

Solvents were dried by recommended literature routes (Burfield *et al.*, 1977, 1978; Burfield & Smithers, 1978). Tris(1-naphthyl)arsine was prepared from arsenic trichloride and 1-bromonaphthalene (Michaelis, 1902). Crystals were obtained by slow evaporation from its chloroform solution.

### Refinement

All hydrogen atoms were positioned geometrically and refined using a riding model with C—H = 0.93–0.98 Å and *U*_iso_(H) = 1.2 *U*_eq_(C).

### Crystal data

**Table:**

C_30_H_21_As·CHCl_3_	*Z* = 2
*M**_r_* = 575.76	*F*(000) = 584
Triclinic, *P*1	*D*_x_ = 1.497 Mg m^−^^3^
Hall symbol: -P 1	Mo *K*α radiation, λ = 0.71073 Å
*a* = 9.1326 (2) Å	Cell parameters from 9912 reflections
*b* = 11.9473 (2) Å	θ = 2.3–35.0°
*c* = 12.3971 (2) Å	µ = 1.66 mm^−^^1^
α = 77.432 (1)°	*T* = 100 K
β = 87.455 (1)°	Needle, colourless
γ = 75.434 (1)°	0.62 × 0.23 × 0.10 mm
*V* = 1277.72 (4) Å^3^	

### Data collection

**Table:**

Bruker SMART APEXII CCD area-detector diffractometer	7382 independent reflections
Radiation source: fine-focus sealed tube	6791 reflections with *I* > 2σ(*I*)
graphite	*R*_int_ = 0.027
φ and ω scans	θ_max_ = 30.0°, θ_min_ = 2.2°
Absorption correction: multi-scan (*SADABS*; Bruker, 2005)	*h* = −12→12
*T*_min_ = 0.427, *T*_max_ = 0.849	*k* = −16→16
37994 measured reflections	*l* = −17→17

### Refinement

**Table:**

Refinement on *F*^2^	Primary atom site location: structure-invariant direct methods
Least-squares matrix: full	Secondary atom site location: difference Fourier map
*R*[*F*^2^ > 2σ(*F*^2^)] = 0.023	Hydrogen site location: inferred from neighbouring sites
*wR*(*F*^2^) = 0.061	H-atom parameters constrained
*S* = 1.04	*w* = 1/[σ^2^(*F*_o_^2^) + (0.030*P*)^2^ + 0.531*P*] where *P* = (*F*_o_^2^ + 2*F*_c_^2^)/3
7382 reflections	(Δ/σ)_max_ = 0.001
316 parameters	Δρ_max_ = 0.47 e Å^−^^3^
0 restraints	Δρ_min_ = −0.29 e Å^−^^3^

### Special details

**Table:**

Experimental. The crystal was placed in the cold stream of an Oxford Cyrosystems Cobra open-flow nitrogen cryostat (Cosier & Glazer, 1986) operating at 100.0 (1) K.
Geometry. All e.s.d.'s (except the e.s.d. in the dihedral angle between two l.s. planes) are estimated using the full covariance matrix. The cell e.s.d.'s are taken into account individually in the estimation of e.s.d.'s in distances, angles and torsion angles; correlations between e.s.d.'s in cell parameters are only used when they are defined by crystal symmetry. An approximate (isotropic) treatment of cell e.s.d.'s is used for estimating e.s.d.'s involving l.s. planes.
Refinement. Refinement of *F*^2^ against ALL reflections. The weighted *R*-factor *wR* and goodness of fit *S* are based on *F*^2^, conventional *R*-factors *R* are based on *F*, with *F* set to zero for negative *F*^2^. The threshold expression of *F*^2^ > σ(*F*^2^) is used only for calculating *R*-factors(gt) *etc*. and is not relevant to the choice of reflections for refinement. *R*-factors based on *F*^2^ are statistically about twice as large as those based on *F*, and *R*- factors based on ALL data will be even larger.

### Fractional atomic coordinates and isotropic or equivalent isotropic displacement parameters (Å^2^)

**Table:**

	*x*	*y*	*z*	*U*_iso_*/*U*_eq_	
As1	0.090367 (13)	0.707273 (10)	0.281681 (9)	0.01270 (4)	
C1	0.23298 (13)	0.55322 (10)	0.33306 (9)	0.0139 (2)	
C2	0.28065 (14)	0.51442 (11)	0.44200 (10)	0.0164 (2)	
H2A	0.2371	0.5585	0.4937	0.020*	
C3	0.39459 (15)	0.40882 (11)	0.47644 (10)	0.0188 (2)	
H3A	0.4246	0.3840	0.5503	0.023*	
C4	0.46095 (14)	0.34296 (11)	0.40176 (11)	0.0188 (2)	
H4A	0.5363	0.2740	0.4251	0.023*	
C5	0.41573 (14)	0.37905 (10)	0.28882 (10)	0.0163 (2)	
C6	0.48349 (15)	0.31226 (12)	0.20993 (11)	0.0214 (2)	
H6A	0.5581	0.2427	0.2327	0.026*	
C7	0.44079 (16)	0.34851 (13)	0.10090 (12)	0.0245 (3)	
H7A	0.4869	0.3041	0.0501	0.029*	
C8	0.32697 (16)	0.45316 (12)	0.06568 (11)	0.0221 (3)	
H8A	0.2985	0.4778	−0.0085	0.027*	
C9	0.25769 (15)	0.51907 (11)	0.14008 (10)	0.0176 (2)	
H9A	0.1815	0.5872	0.1157	0.021*	
C10	0.30027 (13)	0.48509 (10)	0.25366 (9)	0.0143 (2)	
C11	−0.09515 (14)	0.65816 (10)	0.26369 (9)	0.0147 (2)	
C12	−0.09601 (15)	0.54044 (11)	0.27990 (10)	0.0181 (2)	
H12A	−0.0091	0.4825	0.3063	0.022*	
C13	−0.22715 (16)	0.50634 (12)	0.25697 (11)	0.0210 (2)	
H13A	−0.2258	0.4265	0.2690	0.025*	
C14	−0.35565 (15)	0.59027 (12)	0.21725 (10)	0.0206 (2)	
H14A	−0.4410	0.5670	0.2021	0.025*	
C15	−0.36018 (14)	0.71242 (12)	0.19895 (10)	0.0174 (2)	
C16	−0.49212 (15)	0.80103 (13)	0.15651 (11)	0.0225 (3)	
H16A	−0.5780	0.7786	0.1411	0.027*	
C17	−0.49483 (16)	0.91876 (13)	0.13799 (12)	0.0258 (3)	
H17A	−0.5817	0.9756	0.1094	0.031*	
C18	−0.36570 (16)	0.95368 (12)	0.16231 (12)	0.0238 (3)	
H18A	−0.3679	1.0337	0.1499	0.029*	
C19	−0.23685 (14)	0.87058 (11)	0.20412 (10)	0.0179 (2)	
H19A	−0.1531	0.8952	0.2205	0.022*	
C20	−0.22911 (14)	0.74750 (11)	0.22286 (9)	0.0150 (2)	
C21	0.04874 (14)	0.75520 (10)	0.42365 (9)	0.0142 (2)	
C22	−0.06416 (14)	0.72313 (11)	0.49122 (10)	0.0170 (2)	
H22A	−0.1222	0.6782	0.4690	0.020*	
C23	−0.09342 (15)	0.75733 (11)	0.59400 (10)	0.0193 (2)	
H23A	−0.1703	0.7350	0.6386	0.023*	
C24	−0.00874 (15)	0.82332 (11)	0.62790 (10)	0.0193 (2)	
H24A	−0.0278	0.8446	0.6960	0.023*	
C25	0.10744 (14)	0.85955 (10)	0.56059 (10)	0.0167 (2)	
C26	0.19211 (15)	0.93203 (11)	0.59281 (11)	0.0202 (2)	
H26A	0.1735	0.9537	0.6607	0.024*	
C27	0.30057 (16)	0.97039 (12)	0.52556 (12)	0.0226 (3)	
H27A	0.3535	1.0191	0.5473	0.027*	
C28	0.33213 (15)	0.93604 (11)	0.42328 (11)	0.0211 (2)	
H28A	0.4063	0.9618	0.3780	0.025*	
C29	0.25383 (14)	0.86469 (11)	0.39033 (10)	0.0168 (2)	
H29A	0.2772	0.8415	0.3234	0.020*	
C30	0.13786 (13)	0.82569 (10)	0.45661 (10)	0.0143 (2)	
C31	0.01969 (16)	0.16195 (12)	0.04382 (11)	0.0225 (3)	
H31A	0.0208	0.1606	−0.0350	0.027*	
Cl1	0.13525 (5)	0.25237 (4)	0.06542 (3)	0.03896 (10)	
Cl2	−0.16874 (4)	0.21894 (3)	0.08181 (3)	0.03183 (8)	
Cl3	0.08848 (4)	0.01550 (3)	0.11983 (3)	0.02454 (7)	

### Atomic displacement parameters (Å^2^)

**Table:**

	*U*^11^	*U*^22^	*U*^33^	*U*^12^	*U*^13^	*U*^23^
As1	0.01297 (6)	0.01246 (6)	0.01270 (6)	−0.00293 (4)	−0.00088 (4)	−0.00281 (4)
C1	0.0133 (5)	0.0134 (5)	0.0151 (5)	−0.0032 (4)	−0.0005 (4)	−0.0029 (4)
C2	0.0167 (6)	0.0172 (5)	0.0148 (5)	−0.0027 (4)	−0.0009 (4)	−0.0038 (4)
C3	0.0190 (6)	0.0191 (6)	0.0164 (5)	−0.0035 (5)	−0.0036 (4)	−0.0003 (4)
C4	0.0158 (6)	0.0156 (5)	0.0222 (6)	−0.0008 (4)	−0.0023 (4)	−0.0013 (4)
C5	0.0148 (5)	0.0153 (5)	0.0192 (5)	−0.0043 (4)	0.0015 (4)	−0.0041 (4)
C6	0.0184 (6)	0.0192 (6)	0.0267 (6)	−0.0019 (5)	0.0031 (5)	−0.0087 (5)
C7	0.0244 (7)	0.0264 (7)	0.0251 (6)	−0.0045 (5)	0.0056 (5)	−0.0135 (5)
C8	0.0254 (7)	0.0264 (6)	0.0164 (5)	−0.0071 (5)	0.0020 (5)	−0.0080 (5)
C9	0.0192 (6)	0.0179 (5)	0.0154 (5)	−0.0037 (4)	−0.0004 (4)	−0.0038 (4)
C10	0.0133 (5)	0.0151 (5)	0.0151 (5)	−0.0051 (4)	0.0011 (4)	−0.0034 (4)
C11	0.0151 (5)	0.0167 (5)	0.0134 (5)	−0.0052 (4)	−0.0010 (4)	−0.0039 (4)
C12	0.0207 (6)	0.0164 (5)	0.0176 (5)	−0.0057 (4)	−0.0005 (4)	−0.0033 (4)
C13	0.0261 (7)	0.0210 (6)	0.0199 (6)	−0.0122 (5)	0.0022 (5)	−0.0060 (5)
C14	0.0207 (6)	0.0287 (7)	0.0177 (5)	−0.0145 (5)	0.0018 (4)	−0.0069 (5)
C15	0.0156 (5)	0.0258 (6)	0.0120 (5)	−0.0069 (5)	0.0017 (4)	−0.0049 (4)
C16	0.0127 (6)	0.0369 (7)	0.0176 (6)	−0.0062 (5)	−0.0001 (4)	−0.0050 (5)
C17	0.0150 (6)	0.0330 (7)	0.0234 (6)	0.0006 (5)	−0.0005 (5)	−0.0010 (5)
C18	0.0190 (6)	0.0206 (6)	0.0271 (6)	0.0000 (5)	0.0016 (5)	−0.0012 (5)
C19	0.0147 (5)	0.0181 (6)	0.0206 (6)	−0.0033 (4)	0.0003 (4)	−0.0043 (4)
C20	0.0148 (5)	0.0186 (5)	0.0119 (5)	−0.0044 (4)	0.0006 (4)	−0.0034 (4)
C21	0.0156 (5)	0.0125 (5)	0.0143 (5)	−0.0025 (4)	−0.0009 (4)	−0.0033 (4)
C22	0.0182 (6)	0.0152 (5)	0.0180 (5)	−0.0051 (4)	0.0012 (4)	−0.0033 (4)
C23	0.0203 (6)	0.0185 (6)	0.0171 (5)	−0.0029 (5)	0.0041 (4)	−0.0026 (4)
C24	0.0230 (6)	0.0175 (6)	0.0152 (5)	0.0002 (5)	0.0004 (4)	−0.0047 (4)
C25	0.0189 (6)	0.0126 (5)	0.0165 (5)	0.0005 (4)	−0.0040 (4)	−0.0034 (4)
C26	0.0231 (6)	0.0161 (5)	0.0209 (6)	−0.0002 (5)	−0.0078 (5)	−0.0068 (5)
C27	0.0231 (6)	0.0184 (6)	0.0280 (6)	−0.0054 (5)	−0.0097 (5)	−0.0059 (5)
C28	0.0190 (6)	0.0199 (6)	0.0250 (6)	−0.0077 (5)	−0.0046 (5)	−0.0016 (5)
C29	0.0166 (6)	0.0169 (5)	0.0172 (5)	−0.0047 (4)	−0.0017 (4)	−0.0031 (4)
C30	0.0150 (5)	0.0111 (5)	0.0156 (5)	−0.0011 (4)	−0.0032 (4)	−0.0024 (4)
C31	0.0244 (7)	0.0257 (6)	0.0185 (6)	−0.0106 (5)	0.0031 (5)	−0.0028 (5)
Cl1	0.0520 (3)	0.0475 (2)	0.03150 (18)	−0.0357 (2)	0.01156 (17)	−0.01343 (16)
Cl2	0.02568 (17)	0.02956 (17)	0.03121 (18)	0.00074 (13)	0.00267 (13)	0.00334 (14)
Cl3	0.02050 (15)	0.02695 (16)	0.02393 (15)	−0.00309 (12)	−0.00302 (11)	−0.00328 (12)

### Geometric parameters (Å, °)

**Table:**

As1—C21	1.9595 (11)	C16—C17	1.369 (2)
As1—C1	1.9615 (12)	C16—H16A	0.9300
As1—C11	1.9635 (12)	C17—C18	1.411 (2)
C1—C2	1.3808 (16)	C17—H17A	0.9300
C1—C10	1.4326 (16)	C18—C19	1.3717 (18)
C2—C3	1.4147 (17)	C18—H18A	0.9300
C2—H2A	0.9300	C19—C20	1.4219 (17)
C3—C4	1.3678 (18)	C19—H19A	0.9300
C3—H3A	0.9300	C21—C22	1.3762 (17)
C4—C5	1.4197 (17)	C21—C30	1.4362 (16)
C4—H4A	0.9300	C22—C23	1.4154 (17)
C5—C6	1.4191 (17)	C22—H22A	0.9300
C5—C10	1.4267 (17)	C23—C24	1.3696 (18)
C6—C7	1.3688 (19)	C23—H23A	0.9300
C6—H6A	0.9300	C24—C25	1.4159 (18)
C7—C8	1.408 (2)	C24—H24A	0.9300
C7—H7A	0.9300	C25—C26	1.4220 (17)
C8—C9	1.3729 (17)	C25—C30	1.4286 (16)
C8—H8A	0.9300	C26—C27	1.369 (2)
C9—C10	1.4210 (16)	C26—H26A	0.9300
C9—H9A	0.9300	C27—C28	1.4121 (19)
C11—C12	1.3787 (16)	C27—H27A	0.9300
C11—C20	1.4336 (17)	C28—C29	1.3745 (17)
C12—C13	1.4168 (18)	C28—H28A	0.9300
C12—H12A	0.9300	C29—C30	1.4206 (17)
C13—C14	1.367 (2)	C29—H29A	0.9300
C13—H13A	0.9300	C31—Cl1	1.7541 (14)
C14—C15	1.4175 (18)	C31—Cl2	1.7677 (15)
C14—H14A	0.9300	C31—Cl3	1.7681 (14)
C15—C16	1.4219 (18)	C31—H31A	0.9800
C15—C20	1.4262 (17)		
			
C21—As1—C1	98.97 (5)	C15—C16—H16A	119.4
C21—As1—C11	99.78 (5)	C16—C17—C18	119.88 (13)
C1—As1—C11	100.92 (5)	C16—C17—H17A	120.1
C2—C1—C10	119.28 (11)	C18—C17—H17A	120.1
C2—C1—As1	121.43 (9)	C19—C18—C17	120.56 (13)
C10—C1—As1	119.00 (8)	C19—C18—H18A	119.7
C1—C2—C3	121.20 (11)	C17—C18—H18A	119.7
C1—C2—H2A	119.4	C18—C19—C20	121.14 (12)
C3—C2—H2A	119.4	C18—C19—H19A	119.4
C4—C3—C2	120.41 (11)	C20—C19—H19A	119.4
C4—C3—H3A	119.8	C19—C20—C15	118.20 (11)
C2—C3—H3A	119.8	C19—C20—C11	122.68 (11)
C3—C4—C5	120.52 (11)	C15—C20—C11	119.12 (11)
C3—C4—H4A	119.7	C22—C21—C30	119.90 (11)
C5—C4—H4A	119.7	C22—C21—As1	121.23 (9)
C6—C5—C4	121.43 (11)	C30—C21—As1	118.87 (9)
C6—C5—C10	119.22 (11)	C21—C22—C23	121.10 (11)
C4—C5—C10	119.35 (11)	C21—C22—H22A	119.5
C7—C6—C5	121.09 (12)	C23—C22—H22A	119.5
C7—C6—H6A	119.5	C24—C23—C22	120.10 (12)
C5—C6—H6A	119.5	C24—C23—H23A	119.9
C6—C7—C8	119.94 (12)	C22—C23—H23A	119.9
C6—C7—H7A	120.0	C23—C24—C25	120.81 (11)
C8—C7—H7A	120.0	C23—C24—H24A	119.6
C9—C8—C7	120.47 (12)	C25—C24—H24A	119.6
C9—C8—H8A	119.8	C24—C25—C26	121.36 (11)
C7—C8—H8A	119.8	C24—C25—C30	119.58 (11)
C8—C9—C10	121.20 (12)	C26—C25—C30	119.04 (12)
C8—C9—H9A	119.4	C27—C26—C25	121.12 (12)
C10—C9—H9A	119.4	C27—C26—H26A	119.4
C9—C10—C5	118.05 (11)	C25—C26—H26A	119.4
C9—C10—C1	122.70 (11)	C26—C27—C28	120.01 (12)
C5—C10—C1	119.24 (10)	C26—C27—H27A	120.0
C12—C11—C20	119.45 (11)	C28—C27—H27A	120.0
C12—C11—As1	121.68 (9)	C29—C28—C27	120.34 (12)
C20—C11—As1	118.61 (8)	C29—C28—H28A	119.8
C11—C12—C13	121.08 (12)	C27—C28—H28A	119.8
C11—C12—H12A	119.5	C28—C29—C30	121.21 (12)
C13—C12—H12A	119.5	C28—C29—H29A	119.4
C14—C13—C12	120.31 (12)	C30—C29—H29A	119.4
C14—C13—H13A	119.8	C29—C30—C25	118.25 (11)
C12—C13—H13A	119.8	C29—C30—C21	123.25 (11)
C13—C14—C15	120.70 (12)	C25—C30—C21	118.50 (11)
C13—C14—H14A	119.7	Cl1—C31—Cl2	110.57 (8)
C15—C14—H14A	119.7	Cl1—C31—Cl3	110.72 (8)
C14—C15—C16	121.55 (12)	Cl2—C31—Cl3	109.87 (7)
C14—C15—C20	119.34 (12)	Cl1—C31—H31A	108.5
C16—C15—C20	119.11 (12)	Cl2—C31—H31A	108.5
C17—C16—C15	121.11 (12)	Cl3—C31—H31A	108.5
C17—C16—H16A	119.4		
			
C21—As1—C1—C2	−4.02 (11)	C16—C17—C18—C19	0.2 (2)
C11—As1—C1—C2	−105.88 (10)	C17—C18—C19—C20	0.7 (2)
C21—As1—C1—C10	−177.76 (9)	C18—C19—C20—C15	−1.16 (18)
C11—As1—C1—C10	80.38 (10)	C18—C19—C20—C11	178.32 (12)
C10—C1—C2—C3	0.04 (18)	C14—C15—C20—C19	−179.78 (11)
As1—C1—C2—C3	−173.68 (9)	C16—C15—C20—C19	0.66 (17)
C1—C2—C3—C4	0.41 (19)	C14—C15—C20—C11	0.72 (17)
C2—C3—C4—C5	−0.48 (19)	C16—C15—C20—C11	−178.84 (11)
C3—C4—C5—C6	179.76 (12)	C12—C11—C20—C19	179.98 (12)
C3—C4—C5—C10	0.11 (18)	As1—C11—C20—C19	−5.71 (15)
C4—C5—C6—C7	−179.07 (13)	C12—C11—C20—C15	−0.54 (17)
C10—C5—C6—C7	0.58 (19)	As1—C11—C20—C15	173.77 (8)
C5—C6—C7—C8	−0.5 (2)	C1—As1—C21—C22	−87.58 (10)
C6—C7—C8—C9	−0.3 (2)	C11—As1—C21—C22	15.24 (11)
C7—C8—C9—C10	1.1 (2)	C1—As1—C21—C30	92.80 (9)
C8—C9—C10—C5	−1.03 (18)	C11—As1—C21—C30	−164.39 (9)
C8—C9—C10—C1	178.47 (12)	C30—C21—C22—C23	−0.73 (18)
C6—C5—C10—C9	0.19 (17)	As1—C21—C22—C23	179.65 (9)
C4—C5—C10—C9	179.85 (11)	C21—C22—C23—C24	−0.08 (19)
C6—C5—C10—C1	−179.33 (11)	C22—C23—C24—C25	0.82 (19)
C4—C5—C10—C1	0.33 (17)	C23—C24—C25—C26	177.45 (12)
C2—C1—C10—C9	−179.90 (11)	C23—C24—C25—C30	−0.73 (18)
As1—C1—C10—C9	−6.02 (15)	C24—C25—C26—C27	−177.59 (12)
C2—C1—C10—C5	−0.40 (17)	C30—C25—C26—C27	0.61 (18)
As1—C1—C10—C5	173.47 (8)	C25—C26—C27—C28	−1.31 (19)
C21—As1—C11—C12	−105.15 (10)	C26—C27—C28—C29	0.4 (2)
C1—As1—C11—C12	−3.94 (11)	C27—C28—C29—C30	1.25 (19)
C21—As1—C11—C20	80.67 (9)	C28—C29—C30—C25	−1.91 (18)
C1—As1—C11—C20	−178.13 (9)	C28—C29—C30—C21	177.34 (11)
C20—C11—C12—C13	−0.08 (18)	C24—C25—C30—C29	179.21 (11)
As1—C11—C12—C13	−174.21 (9)	C26—C25—C30—C29	0.98 (17)
C11—C12—C13—C14	0.53 (19)	C24—C25—C30—C21	−0.08 (17)
C12—C13—C14—C15	−0.35 (19)	C26—C25—C30—C21	−178.31 (11)
C13—C14—C15—C16	179.27 (12)	C22—C21—C30—C29	−178.45 (11)
C13—C14—C15—C20	−0.28 (18)	As1—C21—C30—C29	1.18 (15)
C14—C15—C16—C17	−179.29 (13)	C22—C21—C30—C25	0.80 (17)
C20—C15—C16—C17	0.26 (19)	As1—C21—C30—C25	−179.57 (8)
C15—C16—C17—C18	−0.7 (2)		

### Hydrogen-bond geometry (Å, °)

**Table:**

*D*—H···*A*	*D*—H	H···*A*	*D*···*A*	*D*—H···*A*
C4—H4A···Cg1^i^	0.93	2.68	3.6013 (14)	169
C14—H14A···Cg2^ii^	0.93	2.86	3.7421 (15)	160

Symmetry codes: (i) −*x*+1, −*y*+1, −*z*+1; (ii) *x*−1, *y*, *z*.
